# Nutritional intervention alleviates T cell exhaustion and empowers anti-tumor immunity

**DOI:** 10.3389/fimmu.2025.1689317

**Published:** 2025-12-19

**Authors:** Yuqi Xu, Wenhui Yuan, Ke Li, Peng Li

**Affiliations:** 1 Guangdong Provincial Key Laboratory of Tumor Interventional Diagnosis and Treatment, Zhuhai Institute of Translational Medicine, Zhuhai People’s Hospital Affiliated with Jinan University, Jinan University, Zhuhai, China; 2 The Biomedical Translational Research Institute, Faculty of Medical Science, Jinan University, Guangzhou, China; 3 Department of Geriatrics, The Seventh Affiliated Hospital, Sun Yat-Sen University, Shenzhen, China; 4 Institute of Cell and Gene Technology, Shenzhen University of Advanced Technology, Shenzhen, China

**Keywords:** antitumor immunity, exhausted T cells, immune checkpoint inhibitors, immunotherapy, nutrients

## Abstract

T cells are central mediators of adaptive immunity, playing a pivotal role in eliminating pathogens and tumor cells. In the context of chronic infections and cancer, however, persistent antigenic stimulation drives T cells into a state of exhaustion characterized by diminished effector function, sustained expression of inhibitory receptors, and profound metabolic reprogramming. Emerging evidence indicates that T cell exhaustion is not irreversible and can be alleviated through immune checkpoint blockade, such as targeting PD-1. Moreover, increasing attention has been directed toward the role of intracellular metabolic pathways in shaping T cell fate and function. Strategies aimed at enhancing nutrient availability and metabolic fitness offer an additional avenue to restore T cell activity. This review highlights recent advances in reversing T cell exhaustion through immune checkpoint inhibitors and nutritional interventions, providing novel insights into the precision and personalization of cancer immunotherapy.

## 1 Introduction

T cells are essential effectors of adaptive immunity and play a critical role in eliminating pathogens and malignant cells. In the setting of chronic infections and cancer, however, persistent antigen exposure drives progressive functional decline, leading to a state known as T cell exhaustion (Tex) (1–4). This dysfunctional state is defined by impaired proliferative capacity, diminished cytokine production, sustained expression of multiple inhibitory receptors, and extensive metabolic reprogramming (5–7). Notably, increasing evidence indicates that exhausted T cells can be functionally reinvigorated through a range of therapeutic strategies, with profound implications for the treatment of chronic infections and cancer (6, 8).

Since Tex is primarily driven by the overexpression of inhibitory receptors, blockade of immune checkpoint receptors represents the predominant strategy to restore T cell function. Immune checkpoint inhibitors (ICIs) can act as potent immune switches, yet their clinical use may be associated with immune-related adverse events (irAEs), particularly when multiple checkpoints are co-targeted (9–14). Moreover, a proportion of patients remains unresponsive to monotherapy with ICIs, underscoring the need for rational combination approaches to enhance therapeutic efficacy.

Beyond immune checkpoint blockade(ICB), novel strategies to reverse T cell exhaustion are rapidly emerging. Within the tumor microenvironment(TME), tumor cells compete with T cells for essential nutrients, thereby restricting T-cell metabolism and accelerating exhaustion. Nutrient supplementation—including amino acids, carbohydrates, fatty acids, and vitamins—has been shown to attenuate the transition of CD8^+^ T-cell toward terminal exhaustion, thereby augmenting their antitumor activity (15, 16). Notably, increasing evidence suggests that nutritional interventions can synergize with ICIs to enhance therapeutic outcomes and inhibit tumor progression.

Effectively reversing the dysfunctional exhausted phenotype remains critical for optimizing immunotherapeutic strategies. In this review, we summarize current knowledge on the role of ICIs and nutritional interventions in reversing Tex, discuss underlying mechanisms, and highlight key directions for future preclinical and clinical research.

## 2 Immune checkpoint inhibitors in reversing T cell exhaustion

**Figure 1:**
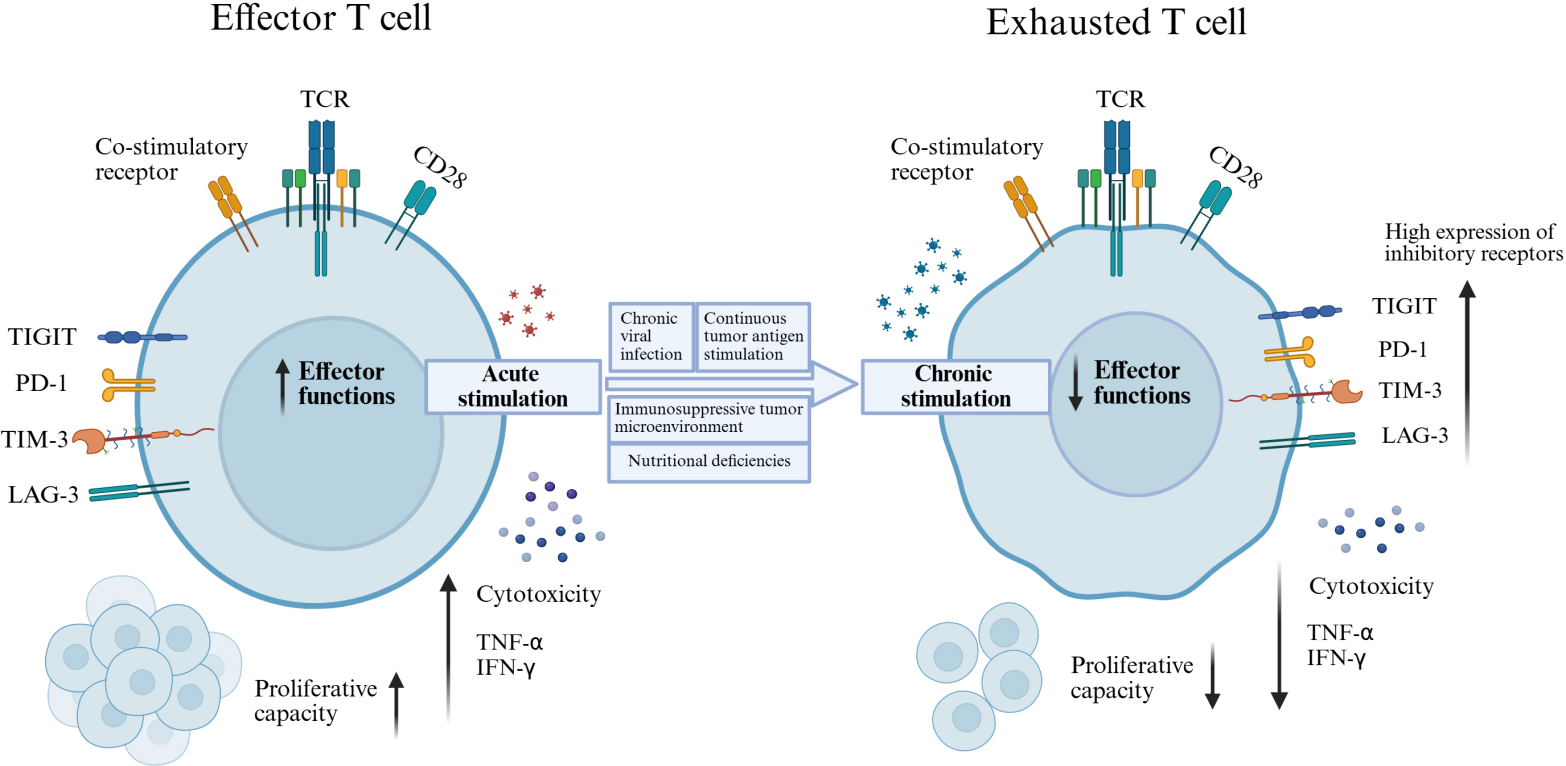
T cell activation versus exhaustion. Acute antigen stimulation promotes full T cell activation, characterized by robust cytotoxic activity, cytokine production, and proliferative capacity. In contrast, persistent antigen exposure during chronic infection or cancer drives effector T cell exhaustion through sustained inhibitory receptor signaling and immunosuppressive cues within the microenvironment.

A hallmark of Tex is the sustained overexpression of inhibitory receptors, including PD-1, T cell immunoglobulin and mucin domain-3 (TIM-3), lymphocyte activation gene-3 (LAG-3), and T cell immunoglobulin and ITIM domain (TIGIT) (17, 18). Under chronic antigenic stimulation, such as in cancer or persistent infection, these immune checkpoints are constitutively expressed on T cells, thereby dampening antitumor immunity (19–21). Immune checkpoint inhibitors can effectively block these inhibitory pathways and restore T cell activity, representing one of the most significant breakthroughs in cancer immunotherapy (22) (**Figure 1**).

### 2.1 Anti-PD-1/anti-PD-L1

Among the inhibitory receptors, PD-1 is the most extensively studied marker of Tex (23, 24). Blockade of the PD-1 pathway partially restores effector function, reduces viral or tumor burden, and has revolutionized immunotherapy (25, 26). PD-1 and its ligands PD-L1 and PD-L2 regulate T cell activation, proliferation, and cytotoxicity in the TME. In particular, PD-1/PD-L1 signaling plays a critical role in establishing and maintaining immune tolerance, thereby restraining antitumor immunity (22).

Monoclonal antibodies targeting PD-1 or PD-L1 have shown remarkable clinical efficacy across multiple cancer types, including melanoma, non-small cell lung cancer (NSCLC), renal cell carcinoma, and bladder cancer (27, 28). Retifanlimab, a humanized anti-PD-1 antibody, has demonstrated durable antitumor activity in melanoma, NSCLC, ulcerative colitis, and Squamous cell carcinoma of the anal canal, supporting its further clinical evaluation in solid tumors (29–31).

Similarly, the anti-PD-L1 antibody atezolizumab improved overall survival (OS) in advanced NSCLC and small cell lung cancer. Furthermore, in the treatment of metastatic colorectal cancer, atezolizumab combined with targeted therapy significantly improved progression-free survival (PFS) and OS (24, 32–34). Collectively, these findings highlight the central role of PD-1/PD-L1 blockade in reinvigorating exhausted T cells.

### 2.2 Anti-CTLA-4

CTLA-4 attenuates T cell activation by outcompeting CD28 for binding to B7.1 and B7.2 co-ligands (35), thereby suppressing proliferation, IL-2 production, and effector differentiation, and contributing to a protumor immunosuppressive environment (36, 37).

Approved ICIs targeting CTLA-4 include ipilimumab and tremelimumab. Ipilimumab enhances adaptive immune responses to tumor cells by blocking CTLA-4 signaling, but its clinical use is limited by irAEs, such as colitis, thrombocytopenia and ICIs-related hypophysitis (38–40). To achieve safer and more effective therapy, novel strategies aim to preserve CTLA-4 signaling in peripheral tissues while selectively promoting Treg depletion or dysfunction within the TME (41). Thus, CTLA-4 antagonism remains a promising therapeutic approach to enhance antitumor immunity (42).

### 2.3 Other immune checkpoint inhibitors

Compensatory upregulation of additional inhibitory receptors, including TIM-3, LAG-3, and TIGIT, frequently occurs after PD-1 or CTLA-4 blockade, leading to acquired resistance (43–47).

Preclinical studies have shown that LAG-3 blockade enhances antigen-specific T cell activation and impairs tumor growth, although limited efficacy is observed with monotherapy. By contrast, dual LAG-3/PD-1 blockade produces synergistic antitumor effects (48–50). Similarly, TIM-3 signaling contributes to Tex and apoptosis in cancer, and its blockade restores T cell proliferation and effector function. TIM-3 is often co-expressed with PD-1, marking a more severe exhausted phenotype (51, 52). Combination therapy targeting TIM-3 and PD-1 significantly enhances antitumor activity and has demonstrated tolerability in early-phase clinical trials (53, 54).

TIGIT is also upregulated in multiple malignancies, including melanoma, NSCLC, and acute myeloid leukemia (55–59). By binding to its ligands, TIGIT directly transmits inhibitory signals to T cells. Preclinical evidence supports TIGIT blockade as an effective approach to reinvigorate exhausted T cells and restore antitumor function (60, 61). These findings provide strong rationale for ongoing clinical trials evaluating dual PD-1/TIGIT blockade, as well as combinations with LAG-3 or TIM-3 inhibitors, in cancer patients.

## 3 Nutritional and metabolic small molecules

**Figure 2:**
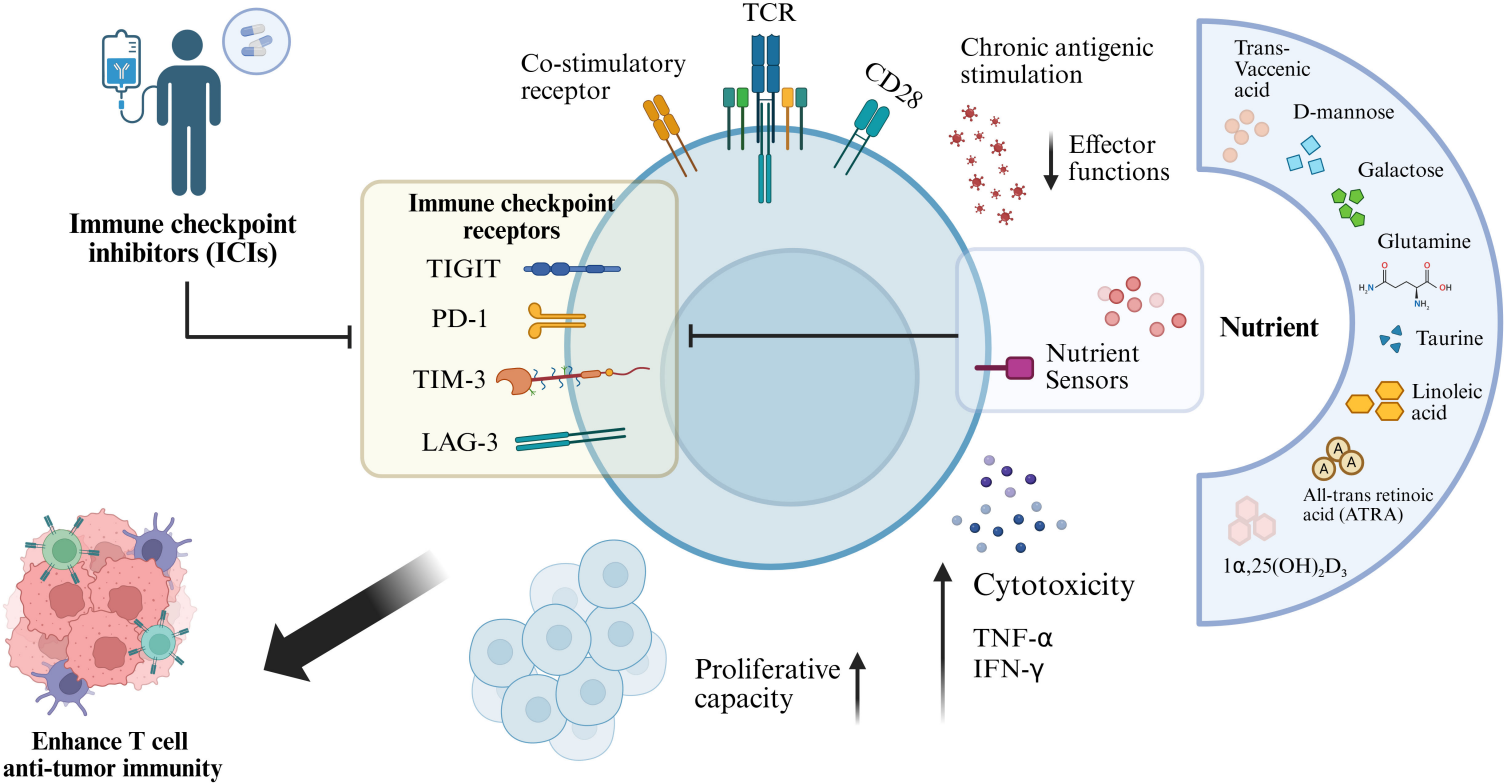
Nutritional intervention reverses T cell exhaustion and enhances antitumor immunity. Chronic antigen stimulation induces T cells to upregulate immune checkpoint receptors, including PD-1, TIM-3, LAG-3, and TIGIT, which results in progressive loss of effector function and exhaustion. Nutritional interventions with key metabolites—such as D-mannose, galactose, glutamine, linoleic acid, and active vitamin D_3_ [1α,25(OH)_2_D_3_]—can modulate T cell nutrient-sensing pathways and metabolic reprogramming. These interventions act synergistically with immune checkpoint inhibitors to alleviate T cell exhaustion, restore proliferative capacity, cytotoxic activity, and effector cytokine (IFN-γ, TNF-α) production, thereby enhancing T cell–mediated anti-tumor immunity.

Nutrient availability is increasingly recognized as a critical determinant of T cell function (62). Deficiencies in essential nutrients can promote T-cell exhaustion through mechanisms distinct from chronic antigenic stimulation. Conversely, supplementation with macronutrients (e.g., carbohydrates) and micronutrients (e.g., vitamins) has been shown to regulate cancer progression, maintain immune homeostasis, reverse Tex, and enhance antitumor immunity (15). Based on a comprehensive review of the current literature, this study further elucidates how nutritional metabolism influences Tex and modulates ICB therapy (**Figure 2**).

### 3.1 Carbohydrate compounds

As fundamental nutrients, carbohydrates are essential for sustaining T-cell activation, proliferation, and effector activity, with their metabolic utilization profoundly influencing the strength and persistence of immune responses. Numerous studies indicate that supplementing specific carbohydrates can remodel tumor microenvironment metabolism, reverse T-cell exhaustion, and enhance responses to ICB. This represents a promising direction for improving cancer immunotherapy (15, 63, 64).

#### 3.1.1 Glucose

The dependence of tumor cells on aerobic glycolysis may be the most extensively studied metabolic adaptation exploited by tumor cells. As research indicates, tumor cells increase glucose uptake by upregulating glucose transporters such as GLUT1 (65). This leads to glucose deprivation in the tumor-infiltrating lymphocyte microenvironment, impairing T cell function. Studies indicate that GLUT1 overexpressing CAR-T cells enhance T-cell glucose competition, improve antitumor efficacy against hepatocellular carcinoma, and increase CD8^+^ T-cell survival rates (65–68). Concurrently, numerous investigations are exploring the combination of glucose metabolism modulation with ICIs (69). For instance, short-term supplementation with high-glucose drinks can enhance T-cell-mediated antitumor immune responses against glioblastoma by modulating the gut microbiota, and exhibits synergistic effects with anti-PD-1 immune checkpoint therapy (70).

In addition, Lactic acid is a byproduct of aerobic glycolysis that can acidify the TME, suppress cytotoxic signaling in CD8^+^ T cells, and upregulate PD-1 expression, thereby enhancing Tex (71–73). Targeting lactate metabolism, such as through monocarboxylate transporter (MCT) inhibitors and LDHA blockers, can alter glycolytic flux in the TME, thereby significantly enhance antitumor immune responses. Furthermore, studies indicate that combining MCT-1 inhibitors with PD-1/PD-L1 blockade therapy can improve the efficacy of ICIs (74–76).

#### 3.1.2 Fructose

High-fructose diets are traditionally associated with metabolic disorders, including type 2 diabetes, cardiovascular disease, and cancer (77). Interestingly, recent studies suggest that dietary fructose can also mitigate CD8^+^ T cell exhaustion and augment antitumor immunity. In mouse models of melanoma and MC38 adenocarcinoma, a high-fructose diet inhibited tumor growth and prolonged survival. Mechanistically, fructose supplementation reduced the accumulation of terminally exhausted PD-1^+^TIM-3^+^ CD8^+^ T cells and improved effector function within the TME (78). Research findings indicate that GLUT5 CAR-T cells maintain potent antitumor activity across multiple tumor models. Importantly, reduced expression of PD-1 and TIM-3 was detected in tumors infiltrated by GT5 OT-I cells. According to these findings, the adoptive transfer of GT5 T cells can be combined with ICIs to enhance their efficacy. (79).

Fructose also regulates adipocyte metabolism, enhancing antitumor T-cell responses through increased leptin signaling (80). Moreover, gut microbiota can convert fructose into short-chain fatty acids (SCFAs), including acetate and butyrate (81), both of which promote CD8^+^ T cell antitumor activity (82, 83). These findings suggest that fructose supplementation may enhance CD8^+^ T cell function via dual mechanisms: elevating adipocyte-derived leptin and increasing microbial-derived SCFAs, thereby attenuating the shift toward terminal exhaustion.

#### 3.1.3 Other carbohydrate compounds

Diminished mannose metabolism is a hallmark of dysfunctional T cells, whereas supplementation with D-mannose restores metabolic programming, preserves stem-like properties through OGT-mediated β-catenin O-GlcNAcylation, and limits exhaustion differentiation. Importantly, D-mannose–expanded T cells retain stemness during long-term culture and exhibit enhanced anti-tumor efficacy, highlighting mannose metabolism as a promising target for cancer immunotherapy (84).

Dietary galactose enhances CD8^+^ T-cell immunity and suppresses tumor progression by reprogramming hepatocyte metabolism to induce IGFBP-1 production, thereby limiting IGF-1 signaling–dependent T cell exhaustion. Elevated plasma IGFBP-1 levels in cancer patients are associated with reduced Tex and enhanced intratumoral T cell responses, highlighting galactose as a potential dietary strategy to improve cancer immunotherapy (85).

### 3.2 Amino acids

Amino acids play a central role in orchestrating T-cell metabolism and function. Their metabolic states interact with, and modulate, the utilization of other key nutrients such as glucose and fatty acids, thereby substantially shaping the development of T-cell exhaustion. Within this framework, we highlight amino acids capable of directly mitigating the exhausted T-cell phenotype and exhibiting synergistic effects with ICIs (15, 65, 86).

#### 3.2.1 Glutamine

Glutamine (Gln), the most abundant free amino acid in serum, is essential for cell growth and proliferation (87). Beyond its well-documented role in tumorigenesis and metastasis (88, 89), Gln is critical for immune cell survival and function. Gln depletion impairs lymphocyte proliferation, reduces expression of activation markers and cytokine production, and induces apoptosis (90). In CD8^+^ T-cell, Gln deprivation not only diminishes effector molecule secretion but also upregulates inhibitory receptors such as PD-1, thereby driving exhaustion (49). Importantly, targeting glutamine metabolism in combination with PD-1/PD-L1 blockade has been shown to restore T-cell function and enhance antitumor immunity, representing a promising therapeutic strategy (69, 91–94).

#### 3.2.2 Taurine

Taurine (Tau) has dual effects in cancer biology, promoting tumor aggressiveness when exploited by malignant cells, yet enhancing CD8^+^ T cell function when available to immune cells (94–96). Uptake of taurine is mediated by the transporter SLC6A6, which is frequently overexpressed in tumors and correlates with poor prognosis. Tumor-driven taurine competition deprives T cells of this nutrient, leading to apoptosis and functional decline (97). Conversely, taurine supplementation rejuvenates exhausted CD8^+^ T-cell, reduces apoptosis, and increases IFN-γ and TNF-α secretion. In preclinical models, taurine administration synergized with PD-1 blockade to enhance antitumor activity and reverse Tex (98).

#### 3.2.3 Other amino acids

L-arginine (L-Arg) is an essential amino acid critical for T lymphocyte function. Arginase I/II(ARG1/2) produced by myeloid-derived suppressor cells (MDSCs) increases arginine consumption in the microenvironment, thereby suppressing CD8^+^ T-cell function and impairing antitumor immunity (99, 100). Inhibiting ARG1/2 within the TME may release T-cell proliferation constraints and activate effective antitumor responses (101, 102). Studies indicate that combining ARG1/2 inhibitors with anti-PD-1 checkpoint inhibitors reshapes the TME, promotes T-cell proliferation, enhances the antitumor efficacy of anti-PD-1 monotherapy, and significantly suppresses tumor growth (101, 103).

Tryptophan deficiency inhibits T lymphocyte proliferation, while indoleamine 2,3-dioxygenase (IDO) inhibitors have been found to activate CD8^+^ T-cell and reduce PD-1 expression by elevating tryptophan levels (104, 105). Research has found that combining IDO inhibitors with ICIs significantly enhances the antitumor efficacy of ICIs (106–108). Therefore, integrating immunotherapy with targeted amino acid metabolism represents a promising strategy for boosting immunotherapy.

### 3.3 Fatty acids

As the third essential pillar of immunometabolism, fatty acids function as both energy substrates and signaling molecules, shaping T-cell activation, proliferation, and survival through uptake or *de novo* synthesis. While dysregulated fatty acid metabolism promotes T-cell exhaustion, emerging evidence indicates that targeted supplementation of specific fatty acids may help restore T-cell function and overcome exhaustion (15, 109).

#### 3.3.1 Trans-vaccenic acid

TVA, a naturally occurring trans fatty acid found in beef, lamb, and dairy products, has been reported to promote tumor infiltration of CD8^+^ T-cell and enhance their cytotoxicity (110). Mechanistically, TVA inactivates the G protein-coupled receptor GPR43, which normally exerts inhibitory effects on CD8^+^ T-cell through cAMP suppression (111, 112). By antagonizing GPR43, TVA activates the cAMP–PKA–CREB axis and enhances CD8^+^ T-cell function. Dietary TVA combined with PD-1 blockade demonstrated synergistic suppression of B16-F10 tumor growth (113).

#### 3.3.2 Linoleic acid

Linoleic acid (LA) has been identified as a positive regulator of cytotoxic T lymphocyte function by promoting mitochondrial fitness and metabolic reprogramming. LA supplementation shifts CD8^+^ T-cell from an exhausted phenotype toward a memory-like state with enhanced killing capacity. (114). This metabolic adaptation improves tumor control, prevents terminal exhaustion, and augments antitumor responses.

### 3.4 Vitamin

Vitamins are essential micronutrients required for numerous physiological processes. Unlike macronutrients such as carbohydrates, proteins, and lipids, vitamins do not provide energy but play critical roles in regulating cellular metabolism and immune function. Recent studies increasingly highlight the importance of specific vitamin supplementation in modulating T-cell activity and enhancing antitumor immunity (115, 116).

#### 3.4.1 Vitamin A

All-trans retinoic acid (ATRA), the active metabolite of vitamin A, functions as an agonist of retinoic acid receptors (RARs) to regulate cell growth, differentiation, immunity, and survival (117, 118). Clinical studies indicate that ATRA enhances responses to ICB by reducing immunosuppression, promoting T-cell activity, and improving prognosis across several cancer types (119).

For instance, co-administration of a RARγ agonist with anti–PD-L1 therapy significantly suppressed tumor growth in an immune checkpoint–resistant lung cancer model. This combination enhanced the proliferation of splenic and tumor-infiltrating T cells, thereby augmenting antitumor immunity (120). Furthermore, dual treatment with ATRA and oxaliplatin markedly potentiated anti–PD-1 efficacy in colorectal cancer models by modulating the tumor immune microenvironment and increasing CD8^+^ T-cell infiltration (119).

#### 3.4.2 Vitamin C

Vitamin C is a crucial micronutrient involved in diverse physiological processes, including immune regulation (115, 121). It has been shown to promote the degradation of PD-L1 protein, thereby reducing its expression and enhancing antitumor immunity (122). Preclinical studies demonstrated that high-dose vitamin C slowed tumor growth in immunocompetent mice. In a lymphoma model, vitamin C synergized with anti–PD-1 therapy, leading to significant tumor inhibition, with some mice achieving complete regression (123). Notably, this combination enhanced CD8^+^ T-cell infiltration more effectively than either treatment alone, underscoring vitamin C’s potential to strengthen immune checkpoint therapy (124).

#### 3.4.3 Vitamin D

Vitamin D, primarily in its active form 1α,25(OH)_2_D_3_, is a fat-soluble vitamin traditionally known for regulating calcium and phosphate homeostasis. Increasing evidence links vitamin D deficiency to elevated risks of cancer and other diseases (125–127), highlighting its therapeutic potential.

Vitamin D can act as an immunoregulatory factor, dampening harmful inflammation while promoting effective antitumor immunity (128). For example, in non-small cell lung cancer patients, 1α,25(OH)_2_D_3_/VDR signaling suppressed inhibitory checkpoint receptor expression (e.g., PD-1) on cytotoxic T cells, thereby enhancing their effector function (129, 130). Moreover, maintaining normal vitamin D levels improved outcomes in advanced melanoma patients receiving anti–PD-1 therapy (131). Mechanistically, vitamin D enhances antitumor immunity partly by shaping the gut microbiota, particularly through promoting the growth of *Bacteroides fragilis*, which synergizes with ICB to inhibit tumor growth (132).

#### 3.4.4 Vitamin B3

Vitamin B3 (nicotinamide and nicotinic acid) is indispensable for cellular metabolism, as it serves as a precursor for nicotinamide adenine dinucleotide (NAD^+^), a cofactor essential for energy metabolism and T-cell function (133). Declines in NAD^+^ impair ATP synthesis, disrupt T-cell receptor signaling, and lead to metabolic dysfunction and impaired T-cell activity.

Pharmacological inhibition of NAD^+^ synthesis, such as via the NAMPT inhibitor FK866, markedly reduces T-cell proliferation and cytokine production (134). Conversely, supplementation with NAD^+^ precursors such as nicotinamide riboside (NR) attenuates T-cell exhaustion and increases CD4^+^ and CD8^+^ T-cell numbers in sepsis mice (135).

Importantly, in preclinical cancer models, NAD^+^ supplementation enhanced the cytotoxicity of CAR-T cells and synergized with anti–PD-1 therapy. CAR-T cells supplemented with nicotinamide (NAM) demonstrated superior tumor-killing capacity, while anti–PD-1 plus NAM treatment delayed tumor progression and improved therapeutic outcomes (133).

## 4 Discussion

Prolonged antigen exposure can trap T cells in a dysfunctional state, while tumor cells activate immune checkpoint pathways to suppress antitumor immune responses (6, 7, 136). Clinically, ICIs (e.g., PD-1/PD-L1 inhibitors) have successfully reversed this exhausted phenotype to deactivate tumors (5, 22, 137). However, only a subset of patients achieve durable responses, as cancer cells activate multiple immune escape mechanisms that limit the efficacy of monotherapy (129, 138).

More recently, increasing attention has focused on how nutrients and small-molecule metabolites can modulate immune checkpoint expression, reverse immune exhaustion, and enhance antitumor responses (15). Combination approaches that integrate ICIs with nutrient-based interventions hold promise for broadening the therapeutic benefit to a larger patient population (91, 123). Given that tumor metabolic plasticity occurs in real time, metabolic intervention targeting a single pathway is highly likely to select for pre-existing or newly acquired drug-resistant cell subpopulations within the tumor. These cell subpopulations not only tolerate treatment but may also accelerate proliferation through compensatory pathways (139). More critically, this may inadvertently induce tumor-infiltrating CD8^+^ T-cell exhaustion, suppress anti-tumor immunity, and promote an immunosuppressive environment. This indirectly provides a sanctuary for cancer stem cells (CSCs), thereby facilitating tumor progression (139–142).

Although it is challenging, we still believe that targeted metabolic intervention in the TME can enhance the anti-tumor immune response. The key lies in dynamically monitoring metabolic changes across different TME cells during treatment and dynamically adjusting metabolic intervention strategies in real time, thereby achieving personalized metabolic tumor immunotherapy. Increasing research indicates that employing isotope tracing (¹³C-glucose/lactate) combined with FDG-PET imaging and magnetic resonance spectroscopy (MRS) to promptly assess tumor metabolism during treatment and adjust metabolic intervention strategies accordingly (140, 143–148).

Additionally, this line of research has offered new insights. By stratifying patients based on cell type-specific metabolic characteristics, it offers a more refined approach to patient segmentation (139). This method holds promise for achieving more personalized and effective cancer treatments, particularly in overcoming drug resistance mechanisms driven by metabolic heterogeneity. Moreover, studies have also revealed cell type–specific nutrient uptake preferences within the TME (149). When designing metabolic interventions, it is important to enhance T cell activation and function by supplying specific nutrients or metabolic modulators, while simultaneously depriving tumor cells and CSCs of key nutrients. This strategy may improve the efficacy of ICIs and strengthen antitumor immunity. Metabolic interventions can also be implemented through cell therapies like GLUT5 CAR-T cells, which precisely target tumor-specific metabolic pathways while avoiding the systemic toxicity associated with metabolic interventions on normal tissues (79).

Looking ahead, the integration of nutritional–metabolic interventions with sensitivity-guided personalized immunotherapy constitutes a key frontier for advancing precise cancer treatment, enhancing clinical efficacy, and reducing therapeutic risks.
